# What do young people with rheumatic disease believe to be important to research about their condition? A UK-wide study

**DOI:** 10.1186/s12969-017-0181-1

**Published:** 2017-07-03

**Authors:** Suzanne Parsons, Wendy Thomson, Katharine Cresswell, Bella Starling, Janet E McDonagh, On behalf of the Barbara Ansell National Network for Adolescent Rheumatology

**Affiliations:** 1 Public Programmes Team, Central Manchester University Hospitals NHS Foundation Trust and University of Manchester, Manchester Academic Health Science Centre, Manchester, UK; 2 Arthritis Research UK Centre for Genetics and Genomics, Centre for Musculoskeletal Research, Division of Musculoskeletal and Dermatological Sciences, School of Biological Sciences, Faculty of Biology, Medicine and Health, Manchester Academic Health Science Centre, The University of Manchester, Manchester, UK; 3 NIHR Manchester Musculoskeletal Biomedical Research Unit, Central Manchester University Hospitals NHS Foundation Trust, Manchester Academic Health Science Centre, The University of Manchester, Manchester, UK; 4 Centre for Musculoskeletal Research, Division of Musculoskeletal and Dermatological Sciences, School of Biological Sciences, Faculty of Biology, Medicine and Health, Manchester Academic Health Science Centre, The University of Manchester, Manchester, UK

**Keywords:** Qualitative, Priority setting, Patient-centred, Patient involvement

## Abstract

**Background:**

The involvement of people of all ages including young people in research is now widely advocated but prioritisation of research topics is still driven largely by professional agendas. Evidence from adult literature has reported a mismatch between a researcher and patient generated list of research topics. There have been no studies to date exploring the priorities of young people with long term conditions other than in SLE. The study aimed to explore the research priorities of young people across the UK with respect to rheumatic conditions.

**Methods:**

Focus groups were undertaken with young people aged 11–24 years with rheumatic conditions recruited across the UK via members of the Barbara Ansell National Network for Adolescent Rheumatology BANNAR and relevant national charities. Data was analysed using a Framework approach. Participants discussed their beliefs about what should be researched in: Basic Science; Clinical Medicine; Health Services, Psychosocial, and Public Health. They were then invited to prioritize these areas in terms of how much funding they should receive.

**Results:**

Thirteen focus groups were held involving 63 participants (18 males: 45 females, mean age 16 years, range 10 to 24) in all four nations of the UK. Young people’s research priorities were influenced by whether they felt research would achieve benefits for all or just some patients and long or short term goals. Another influence was whether participants felt that research areas were already well funded.

Across all groups, Basic Science was a key priority and participants felt that psychosocial research should be prioritized more. Health Services Research was a lower priority, as the majority of participants were happy with their care. Clinical medicine was not a high priority as young people were happy with their medication or uncomfortable with trying new ones. Finally, for nearly all groups, Public Health was a low priority. Differences were also observed between the two age groups and across the geographically diverse focus groups.

**Conclusion:**

Understanding young people’s research priorities is important to develop research that is in tune with their needs. The results highlight the importance of considering the whole age range of adolescence and young adulthood as well as geographical diversity. The findings from this work will inform the future research of the Barbara Ansell National Network for Adolescent Rheumatology BANNAR in the UK.

## Background

Involving people at all stages of health research (including young people) is now widely recommended, considered to be ethically important and has been called for by young people themselves [1–3]. In the UK, this has resulted in changes to the grant and ethics committee processes and to patient and public involvement (PPI) being widely advocated by professional bodies. For example, the UK Royal College of Paediatrics and Child Health have recently developed a research charter with, and for, children and young people [4].

A key area where patients and the public can have an influence is in setting research priorities. However, until recently, prioritization of research topics has been led by professional agendas rather than the public’s views [5]. In the UK, the James Lind Alliance has led the way in involving patients in research priority setting, with the development of priority setting partnerships (PSPs). PSPs bring together patients, carers and clinicians to identify and prioritize the “unanswered questions” about their treatments [6]. However, there are few studies that purely include young people in research priority setting. Previous priority setting exercises have tended to involve either a small number of young people alongside adults [7], or adults acting as a proxy for young people’s views. Therefore, there may still be a risk that the limited research funding available may be directed to research topics which young people do not value as highly as researchers or adults. It is also not clear whether research priority setting partnerships involving young people subsequently impact on the research agenda.

The Barbara Ansell National Network for Adolescent Rheumatology (BANNAR) is a network of research interested rheumatology professionals that focuses on ensuring that every young person in the UK has the best chance to benefit from developments in the field of adolescent and young adult rheumatology [8]. A key objective for BANNAR is involving young people in developing the research priorities for the network and into future projects conducted by BANNAR members.

The study aimed to explore the research priorities of young people across the UK with respect to rheumatic conditions.

## Methods

The study protocol has been published elsewhere [9]. In summary, this was a qualitative study of young people with rheumatic conditions. 16 focus groups were planned across the UK (8 with 11–15 years olds and 8 with 16–24 years olds). The age ranges were chosen to reflect adolescent developmental stages i.e. early and mid-adolescence (11–15 years) and late adolescence and young adulthood (16–24 years) [10].

To ensure national representation, focus groups were conducted in all four nations of the UK. This strategy helped to reflect the differences in health service organization and the impact this may have on young people’s beliefs. We decided at the outset to undertake focus groups in all four nations even if data saturation had been reached. This was to ensure the success and inclusivity of the ongoing involvement strategy for BANNAR [8].

### Inclusion criteria

English speaking young people aged 11**–**24 years old and under the care of a rheumatologist for a range of chronic conditions including: inflammatory arthritides (juvenile idiopathic arthritis, inflammatory bowel disease associated arthritis, adult rheumatoid or psoriatic arthritis, ankylosing spondylitis) in addition to connective tissue diseases (such as SLE, scleroderma, vasculitides), chronic recurrent focal osteomyelitis, and chronic idiopathic pain syndromes. In view of the impact of pain during adolescence irrespective of condition [11] and the significant proportion of young people with chronic idiopathic pain syndromes seen in rheumatology practice in the UK, the latter were included in this study. The emphasis of the study was to ascertain the opinion of young people regarding chronic rheumatic conditions, inflammatory and non-inflammatory rather than condition specific issues.

Exclusion criteria included young people not under the care of a rheumatologist and/or insufficient spoken English to participate independently.

### Recruitment

Senior rheumatology team members provided a broad range of eligible young people (in terms of their age, gender, ethnicity, condition experienced, prior research experience and socio-economic status) with a study information sheet, and the contact details of the research team. Young people were also recruited via Arthritis Care (a UK based national charity) to ensure that the study involved those who were under the care of rheumatologists not associated with BANNAR [12].

Over 16 s gave their individual consent to participate and 11–15 years olds gave their assent following parental consent. Parents of those aged 11–15 were able to accompany participants but waited in a separate room whilst their child took part.

Focus groups were moderated by SP (a social scientist) and/or JMcD (a Paediatric Rheumatologist) who had no direct involvement in the clinical care of participants in order to limit researcher bias. Both moderators were experienced in focus group methodologies and therefore acknowledged existence of the following beliefs (i) young people with rheumatic disease have research priorities that are unique to their developmental status; (ii) that rheumatic disease influences these priorities and (iii) their personal research priorities in addition to experience of research with young people. The researchers considered how such personal beliefs and experiences may bias interpretation of the data and set these aside during moderation of the groups, analyses and writing up of results.

Groups were held at easily accessible locations and all expenses were covered for participants and their parents. Participants received a £20 gift voucher to thank them for taking part.

### Focus group topic guide

Focus groups were designed to be as interactive as possible. Discussions lasted up to 90 min. Further details of the focus group topic guide are available in the protocol paper [9]. Approximately 45 min was allocated to exploring the research priorities for young people. To do this, the Research team briefly described the areas which are currently researched in rheumatology: ***Basic Science; Clinical Medicine and Science; Psychosocial; Health services; Public Health*** [13] – Table 1.

**Table 1:** Research priority areas discussed within the focus groups

Research priority areas
***Basic Science*** - *Research that tries to increase our understanding of rheumatic disease and of finding a cure. Results may not have immediate or direct benefit to patients*
***Clinical Medicine and Science*** – *Research focusing on finding better treatments for rheumatic disease*
***Psychosocial*** – *Research which focuses on improving physical, psychological, social and spiritual outcomes for young people diagnosed with rheumatic disease and their families*
***Health services*** – *Research that explores the best way to organise, manage, finance and deliver care to young people with rheumatic disease and their families*
***Public Health*** – *Research which focuses on putting policies in place, laws or population wide measures to improve outcomes for young people with rheumatic disease and their families*

When discussing each research area, participants were invited to give their ideas regarding what it is important for researchers to focus on. After discussing the areas, the groups were asked to rank them from those which they believed should receive the most funding (score 1) to those that they believed should receive the least (score 5). Whilst doing this they were asked to discuss the rationale for their choices.

Additional data was collected addressing whether and how young people would like to be involved in research and this will be reported elsewhere.

### Data management

All recordings were transcribed verbatim and then pseudonyms were created for names, organisations and places. Thematic data analysis was carried out using the Framework approach. This required the research team to develop a thematic framework from the transcripts by mapping the interviewees’ ideas and collapsing them into themes and sub themes. The thematic framework was then applied to the data. The Framework approach to qualitative data analysis was chosen because of its transparent nature and utility within a research team and its strength in facilitating between and within case analysis [14]. Framework also allowed both a priori and emergent themes to be included within the analysis. SP, JMcD and WT developed the thematic framework, the framework was then applied to the data by SP and KC and the data analysis was then discussed by all.

Data was analysed by SP and KC.

## Results

In total, 13 focus groups were held across the UK (England 8; Scotland 2; Northern Ireland 2; Wales 1). Six focus groups were held with 11–15 years olds (*n* = 30) and seven with 16–24 years olds (*n* = 33). Participants’ ages ranged from 11 to 24 years (mean = 16). As the aim of the study was to explore the research priorities of young people with a broad range of rheumatological conditions rather than condition-specific research questions, details of the individuals were not collected beyond recruitment in accordance with the ethics approval. The moderators of the groups however can confirm that the majority of young people had JIA or SLE.

Further details of participants’ characteristics are available in Table 2.

**Table 2:** Characteristics of participants

	UK	England	Scotland	Northern Ireland	Wales
Age group					
11–15 years olds	30	20	5	5	0
16–24 years olds	33	19	2	9	3
Gender					
Male	20	15	2	3	0
Female	43	24	5	11	3

The following themes were identified from our analysis

- Participants’ beliefs about which research areas should be prioritized over others
- Participants’ beliefs about what should be researched within each area
- The influences on participants’ research priorities

### Participants’ beliefs about which research areas should be prioritized over others

Tables 3, 4, 5 details how the groups of young people ranked the research areas from those which they believed should receive the most funding (score 1) to those that they believed should receive the least (score 5). Ranks could be equal.

**Table 3:** Rankings of research priorities by individual focus groups of under 16 years olds

	Research area				
Focus Group	Basic Science	Clinical Medicine and Science	Psychosocial	Health Services	Public Health
1	2	1	3	4	5
2	1	4	2	3	5
3	1	3	1	3	5
4	1	3	2	5	4
5	5	2	2	4	1
6	1	3	1	4	4
Average rank – Under 16 s	1.8	2.7	1.8	3.8	4

**Table 4:** Rankings of research priorities by individual focus groups of 16 + years olds

	Research area				
Focus Group	Basic Science	Clinical Medicine and Science	Psychosocial	Health Services	Public Health
7	5	2	1	4	3
8	3	1	2	5	4
9	4	1	2	3	4
10	1	3	2	5	4
11	1	2	4	3	5
12	2	5	1	3	4
13	3	5	4	2	1
Average rank – Over 16 s	2.7	2.7	2.3	3.6	3.6

**Table 5:** Total and average rankings of research priorities by individual focus groups of 11–24 years olds

	Research area				
Focus Group	Basic Science	Clinical Medicine and Science	Psychosocial	Health Services	Public Health
Total of rankings – all groups	30	35	27	48	49
Average rank all groups	2.3	2.7	2.1	3.7	3.8

### Participants’ beliefs about what should be researched within each area

#### Basic science

In both age groups (11–15 years and 16–24 years) Basic Science was considered a key priority (Tables 3, 4, and 5).

Participants believed that the ultimate aim of this area should be to find a cure for their conditions (Table 6). However, they also expressed varying degrees of confidence as to when and how this might happen. In general though, participants believed that finding a cure was unlikely to happen overnight, and therefore providing a steady stream of funding to Basic Science over a long time period was likely to be the most appropriate approach to funding.

**Table 6:** Participants’ Beliefs regarding Basic Science Research

Priority	Example quotes
Finding a cure	*F: Well curing it is important; it is obviously going to affect a lot of people if you can actually cure it, because there’s always a chance of it coming back. So to cure it would definitely be the bes*t. (England 11–15)
Understanding the genetic basis of conditions	*F: But when I asked them they said there could be a genetic link but then my mum doesn’t have it, and my dad doesn’t have it, no one, it doesn’t run in the family. So where did it come from?* (England 11–15)
Understanding the relationship between a patients’ genetics and their treatment	*F- I’d agree with that because they will put on medicine that you may get side effects or something like that, and then you complain about them and they say ‘oh they are just headaches. So even if they could look at the person, no one’s ever looked at my genetics, even if they did that little bit of extra research into their patient.* (England 16–24)
Development of predictive tests	*F- So if there was a test to find out how much it was going to progress, and also which drugs work best with a young person. (England 11–15)*

*F* Female, *M* Male; Country focus group undertaken in; age group

Participants expressed a great deal of interest in research to increase understanding of the genetic basis of their conditions. Participants were particularly interested in research focusing on understanding individuals’ response to medication (including side effects and disease response) and on developing predictive tests to help predict the progression of their condition in the future.

#### Clinical medicine

For many, the development of new treatments was a low priority (Tables 3, 4, and 5). Potential reasons for this were that they were happy with their current treatment regime, or they were uncomfortable with the perceived risk of trying a new and experimental treatment. Those who were less satisfied with their treatment experience appeared to have a greater interest in trials of new treatments.

Initially some participants appeared to believe that randomized controlled trials could just be of new medicines. However, once the moderator explained that all treatments could be trialed included talking therapies, participants’ interest in clinical medicine increased. As well as trials of talking therapies, another area of interest for participants was undertaking trials of improvements to how medicines are administered, e.g. less painful ways of giving injections (Table 7).

**Table 7:** Participants’ Beliefs regarding Clinical medicine research

Priority	Example quotes
Trials of new formulations of existing medicines	*M- I have methotrexate and humira, and I really, really get sick when I have my methotrexate. So I would probably try and develop one that doesn’t make you sick that is not chemotherapy based. (England 11–15)*
Trials of new medications	*M- Your body starts developing antibodies against it and methotrexate knocks your immune system off so it doesn’t start developing antibodies. But I wish there was another source of doing that. So they need to find another route other than methotrexate and steroids (England 11–15)*
Trials of psychosocial therapies	*F- But if there was to be things like talking therapies and everything then you get the chance to explain other ways that it actually affects you. You actually get to talk about how it affects, makes you feel and stuff like that. (England 16–24)*
Differences in researchers and patients overall priorities	*F – Medication might be an important one we don’t like to take methotrexate because it makes us sick. Researchers are looking at what methotrexate does to joints. And where our thing is is that we are having to take them so we want that to be the priority. (Wales 16–24)*

*F* Female, *M* Male; Country focus group undertaken in; age group

#### Psychosocial

Overwhelmingly, participants felt that psychosocial research doesn’t receive enough investment (Tables 3, 4, 5 and 8. Those who felt that the psychosocial aspects of their condition had been neglected, or that they had inadequate access to psychosocial support services tended to prioritise this area more than those who felt well supported psychologically or not affected by their condition in this way.

Participants were particularly interested in research which explored the best ways of providing support to recently diagnosed young people (a time when little support was felt to be available). They were also interested in how their families could be better supported, as they were acutely aware of the impact their health had on their parents and siblings. Providing better support for parents was felt to be especially important to help parents to understand and better support their children’s needs.

When discussing this area, participants regularly mentioned the Childhood Health Assessment Questionnaire (CHAQ), a questionnaire regularly used in rheumatology research and treatment [15]. The participants felt it was too long and did not capture their experience of their condition. Finally, the psychosocial impact of transition from paediatric to adult care was identified as a key priority area when discussing psychosocial issues. Some participants had already made the transition from paediatric to adult care, with some reporting positive experiences especially if they had access to a transition coordinator. However, others reported feeling very unsupported and affected by their move into adult care (Table 8).

**Table 8:** Participants’ Beliefs regarding Psychosocial research

Priority	Example quotes
Peer support	*F- I think when you are first diagnosed; it should be maybe someone over 15 or over 18. I think there should be someone who comes and explains it to you. Because you’ll listen to someone who’s roundabout your age. But having someone who’s actually been through it and not whose just helped patients. (England 11–15*)
Support for parents and siblings	*F- So I don’t know if it would be like classed as the research but I think the funding needs to be not just for the kids but for the entire family to talk to someone because it’s not exactly a nice thing to go through. (England 11–15)*
Transition from paediatric to adult care	*F –Just because of the position I’m in as well, because I’ve got to the stage where I’m going to have to probably move on to adult services. I am terrified of that transition. There’s nothing put in place to kind of the ease that whole transition. (Northern Ireland 16–24)*
Assessment of psychosocial aspects of their rheumatic condition	*F: When I go you have to say how you were in the past week, but I might have been completely fine in that past week but maybe before I was in a lot of pain, but you can’t note that down so they can’t completely understand what it’s been like. (England 11–15)*

*F* Female, *M* Male; Country focus group undertaken in; age group

#### Health services research

Health Services Research was considered a low priority by many participants (Table 3). One explanation given for this was that they were happy with how their treatment was being organized and so they did not feel that it needed to change. For many, consultant care or care from a multi-disciplinary based hospital team was the preferred option, especially if they had easy access to hospital care. Receiving more care from Family Physicians (General Practitioners (GPs) in the UK) was not considered a viable option. This was mainly due to participants’ reported poor experiences (particularly when trying to reach an initial diagnosis for their condition) which appeared to have negatively affected their views of GPs as a treatment option.

However, those without easy access to hospital care tended to be more open to being treated by their GP in the future and were interested in research that explored how to improve communication between primary and secondary care and in more effectively involving GPs in their management (Table 9).

**Table 9:** Participants’ Beliefs regarding Health Services Research

Priority	Example quotes
Reliance on specialist care	*F: I think it’s quite an important area because I would much prefer to be looked after under the specialist services, because they’ve really focused in that area and they know much more about it. Whereas when I was been diagnosed, when they were trying to find out what was wrong. I will go to the GP and they wouldn’t really know. It took me like 2 years because they were saying it will just be that (England 11–15)*
Interest in other models of care and services	*F- If GPs knew more about the ways to treat it and things then lots of young people would be better treated by their GPs. Cause it’s much nearer to their homes. So like for me it takes an hour to get to the hospital..It would be much better if your GP could manage things for you (England 11–15)* *F6- I think you should put more funding into putting children’s hospitals all of the country instead of just one in like the west midlands. For me it’s easier to get to the hospital because I live in Birmingham, but for others it’s like they are travelling for miles, going on a train (England 11–15)*
Learning from good practice in terms of health services	F: So maybe that’s for certain areas and not for everyone. Or maybe it is just learning the ways of how others, like X is good for that multi-disciplinary team, so the research is done into how that can be extended into other places, more than new research being done, just utilizing what is already there. (Wales 16–24)

*F* Female, *M* Male; Country focus group undertaken in; age group

#### Public health

For nearly all groups, Public Health was ranked low in their priorities (Tables 3, 4 and 5). This appeared to be because participants believed that other research areas would have a greater impact on their individual health and also because participants in some cases were skeptical as to whether the wider public’s beliefs about their conditions could be changed.

Nevertheless, a number of participants spoke at length about the difficulties they experienced in having their needs understood at both school and in the workplace and how raising public awareness that young people can have rheumatic conditions could help with this (Table 10).

**Table 10:** Participants’ Beliefs regarding Public Health Research

Priority	Example quotes
Raising awareness of condition in schools and workplaces	*M- I think in school they need to be like more educated because at first the school just thought I was wagging it off but then I brought in the medical letters from the hospital and then still didn’t believe me. (England 16–24)*
Raising awareness of invisible conditions	*F- I heard on the news a little while ago something about a disabled person with arthritis who couldn’t walk upstairs or something, wanted to use the disabled toilet. And they were told that they weren’t allowed because they didn’t look disabled enough. So I think they kind of need to raise awareness in that sort of thing. (England 11–15)*
Developing patient databases/biobanks	*M- If you know, you are in a rural area with one local hospital and the doctor there has only heard of Lupus from a medical drama or something. It would be really helpful for them if they could go and get general statistics. What’s the general symptoms look like and how if this worked well or that worked well. So better communication between these large scale research projects given to more healthcare professionals you know could improve treatment. (England 16–24)*

*F* Female, *M* Male; Country focus group undertaken in; age group

### The influences on participants’ research priorities

Young people’s priorities varied across the groups with their decisions appearing to be related to factors that were:

1. Specific to them as an individual,
2. Specific to the wider community of young people with rheumatic conditions
3. Influenced by their existing beliefs about rheumatology research (Table 11).

**Table 11:** Influences on young people’s research priorities

Influences on research priorities	Example quotes
Benefits on an individual or population level	*F4- Well we thought it was important to find a cure otherwise it will keep on happening for generations and also if someone has it worse than another person then we need to figure out why. (England 11–15)*
Extent to which the area affects you	*M2- Luckily my condition doesn’t affect me that much. You know in the lupus group we go to there are people there in wheelchairs for years, missed two to three straight consecutive years of school. But I am practically normal in comparison to some people with this condition. But if I am to take a constructive approach then it is definitely underlooked. (England 16–24)*
Beliefs about the extent to which research areas are already funded	*Basic Science* *F: Probably yes I reckon quite a lot of people find it important and find out more about it so. So I presume it is getting a lot of money. (England 11–15)* *F: I think finding a cure would probably get quite a lot of funding personally, but I think the bits we say about side effects I don’t think they necessarily would. (England 16–24)* *Psychosocial* *I think it’s something that’s definitely overlooked, because in a way I suppose people just expect you to get on with it and manage it, obviously it’s hard for everyone, most people can, but it’s not something that’s really looked at. It’s like a touchy subject or something England 16–24*
Beliefs about the amount of funding research requires	*So for things like arthritis they probably don’t put money into it because they think they know the best way to treat it already. They just think, this has worked for somebody else, and then this will work for everyone. This isn’t really the right attitude to have but* (England 16–24 (Clinical medicine)

*F* Female, *M* Male; Country focus group undertaken in; age group

For most of the research areas, participants discussed whether the key research issues were problematic for a large or small number of patients. Therefore, if a research area was believed to be essential to focus on and problematic for a large number of those with rheumatic conditions, then it was believed to be more important to fund than if it produced large benefits but for fewer patients.

Participants also weighed up the personal impact of focusing on a particular research area, with the wider population impact, and in the majority of cases prioritized an area if it was likely to impact on a population basis.

Participants also considered whether funding research areas would help young people achieve short or long term health goals, for example, finding a cure in the future, versus improving quality of life now. Participants appeared divided in whether they believed priority should be given to short term or long term goals, and this appeared to be related to how long participants had been experiencing symptoms for.

Finally, participants’ beliefs about whether a research area was already well funded influenced whether they felt it should be prioritized.

## Discussion

The current study is the first nationwide to specifically consider the research priorities of young people (including those in early adolescence) with a range of long term rheumatic conditions. A recent study by Tunnicliffe et al. reported research priority setting in young people (from mid adolescence to young adulthood) with SLE in one region of Australia [16].

The current study provides evidence that young people (including those in early adolescence) are able to articulate their views about research even if they are relatively research naïve. Research with the latter age group is theoretically more challenging as development of abstract cognition and hence conceptual thought has been found to be delayed in some young people with chronic illnesses. [17]. In practice, young people in early adolescence are less often invited to contribute to the early stages of research projects and we therefore were keen to involve the 11–15 years olds in the current study.

When comparing the accounts of the 16–24 years olds and the 11–15 years olds, many of the same issues were raised in both groups, particularly in relation to young people’s experience as patients and their concerns in communicating their condition to the wider world. However, as is to be expected, the 16–24 years olds were in many cases talking about past experiences and challenges rather than issues they were currently experiencing. The older age groups were also more likely to talk about work and higher education and their experiences of transferring from paediatric to adult care. Despite these differences there was still a great deal of similarity between the issues raised in the younger and older age groups. This may have been related to both age groups having considerable experience of their condition.

Understanding how young people form their opinions about research will be important when considering how to most effectively engage and involve young people in future studies. Their own personal experience as a patient and the information seeking undertaken by both themselves and their families regarding the causation and management of their condition are potential influences. Likewise the media and the school curriculum may also influence young people’s opinions. Understanding the range of influences on young people’s beliefs about research is an area worthy of further study.

Interestingly, young people also expressed opinions on the extent of current funding of areas, i.e. the perception that psychosocial research is currently underfunded, although it remains unclear as to what is informing these views. One explanation may be young people’s experience of care, i.e. that they did not feel certain aspects of the care were currently heavily focused on.

### Beliefs about specific research areas

For the majority of groups interviewed, Basic Science was considered to be an important area, as ultimately they wanted to be free of their conditions but at the same time acknowledged that a cure was unlikely to be found overnight.

Young people expressed great interest in genetics, both in terms of whether their genetics could explain why they had a rheumatic condition and also whether a better understanding of their genetics could increase the acceptability of treatment. These findings suggest that the young people interviewed may need further support to understand complex genetic disease. Potential explanations for this interest could be increased knowledge via the media of personalized medicine as well as the recent inclusion of genetics into the UK school curriculum. This belief could also have resulted from young people’s significant personal experience of receiving treatments which caused side effects and didn’t appear tailored to their individual needs.

Psychosocial research was perhaps the research area which provoked the most discussion from young people. Gaining a greater understanding of the psychosocial impact of rheumatic conditions for adolescents and young adults will be an important area for future research, a finding echoed by previous research involving young people with SLE in Australia [16]. This research may include the assessment of psychosocial impact by rheumatology professionals in addition to effective interventions in the context of routine clinical care. Psychosocial research was also an area where young people felt that they could potentially play an active role in providing support to others. Acknowledging the expertise of young people in living with and managing their rheumatic condition is an important skill for both clinicians and researchers alike [18]. Recent research has focused on the role of peer support in rheumatology [19]. A recent systematic review highlighted the need for further research on how to provide greater reach and adoption of peer support interventions using digital technologies [20].

A particular practical issue of research with young people is the availability of developmentally appropriate measures. In the current research, issues regarding the CHAQ [15] were raised by a number of participants. The CHAQ, although validated for use by young people up to the age of 18, is written in the third person for a parent proxy report. Young person centred outcome measures (written in the first person) are increasingly available and any future research in this area should ensure involvement of young people in development of measures in order for them to be both acceptable to young people as well as accurately capture their experiences of their condition was identified as an important priority. Irrespective of which measure is used, consideration of the developmentally-appropriate application of these measures in clinical practice remains important eg explanation of the rationale for use.

Finally, psychosocial research was an area where participants also prioritized support for their family and friends. A recent systematic review has reported the challenges faced by parents of young people with long term conditions during their transition from paediatric to adult care [21]. Even if individuals did not feel that their condition affected them psychosocially, they had an acute awareness that some people were very affected in this way and that additional and improved support may be required. Such altruistic reasoning has been studied previously in other areas [22, 23], although it has also been noted that researchers cannot always assume that altruism will lead directly to research participation [24].

When discussing clinical medicine research, a number of young people described poor experiences of attempting to find the ‘right’ medication for them, in terms of being both effective and not causing side effects. This previous experience and concern about taking a risk on a new medicine may have influenced their beliefs about clinical medicine research. Young people’s motivations to take part in clinical trials have recently been explored in a qualitative UK study, albeit not specific to rheumatology, in which young peoples’ motivations to take part in clinical trials were both personal benefit and helping others although both these incentives were more complex than expected [22]. Gaining a greater understanding of young people’s perceptions of the risk of taking part in trials in the future may be useful in increasing both participation but also young people’s understanding of what they are taking part in. These findings support the need for a greater understanding of young people’s decision making regarding clinical trials participation, including their perceptions of risk. The development of improved information on randomized controlled trials for young people and their families is also likely to be beneficial particularly as there is existing evidence to suggest that the adult population’s knowledge and awareness of RCTs is low [25].

When discussing health service research as a research area, the majority of young people interviewed were still wedded to receiving care from their specialist hospital based team rather than considering other treatment options like the GP. This reluctance to change the status quo is likely to have impacted considerably on the extent to which they prioritized health service research. It may be challenging to change this view, particularly of GP care, as many participants reported poor experiences with their GPs especially prior to their diagnosis which appeared to have persisted. Unfortunately recent UK research has failed to show a reduction in time from onset of symptoms to referral to a paediatric rheumatologist over the last decade for children and young people with a subsequent diagnosis of juvenile idiopathic arthritis [26]. The focus on secondary care obviously has considerable implications in terms of health service costs. Exploring this finding in more depth and working with young people to design new models of care closer to home is likely to be important in the future.

Public Health was ranked low in the list of priorities by many of the groups. This may have been due to public health being a broad reaching area which was challenging for facilitators to explain and perhaps for the groups to understand. It may also have been because participants felt that focusing on other areas may have improved both their and others health and quality of life more. However within Public Health, a key area of interest for all participants was raising awareness of their conditions amongst the public in general and in schools and workplaces. Several authors have recently highlighted the need for further study of this area in this particular age group [16, 27, 28].

### Strengths and limitations of this study

A strength of this study, was the inclusion of 63 young people from a broad range of ages and from all 4 UK nations, thereby reflecting a range of adolescent and young adult rheumatology service provision. The variation in opinion was an important finding, validating our approach and highlighting the need to involve a varied sample in planning research projects*.* Research areas which had more variation included clinical medicine and health services research with those young people satisfied with their care and management prioritizing these areas less than the others.

As a convenience sample was recruited there is potential for selection bias. However, qualitative research aims not to produce a statistically representative sample, but instead to create a non-random sample (maximum variety purposive sample) reflecting the diversity of a given population. In addition to requesting participating teams to recruit a broad range of eligible young people, young people were also recruited via a national charity to ensure that the study involved those who were under the care of rheumatologists not associated with BANNAR. Furthermore we did reach data saturation before completion of all the planned focus groups although did continue in order to involve all 4 nations of the UK.

Recruitment for this study was poor in some areas particularly if there was not a Public and patient Involvement (PPI) coordinator or transition coordinator who could help with recruitment. In the development phase of this research, a survey of the 25 main paediatric rheumatology units identified a team member with PPI included within their job description in only 5 units [9]. Challenges in recruitment may also have reflected variations in research culture in rheumatology across the UK.

Given the recruitment challenges in addition to the UK nature of the study, the results are of potential limited generalisability, particularly to populations where lack of access to appropriate healthcare and awareness are not necessarily available. However, we did consider the 4 nations of the UK which differ in paediatric rheumatology provision and recruitment was conducted by both hospital based teams in addition to a national charity.

Within these focus groups, many participants described low levels of awareness and experience of research. For this reason we discussed their experiences as patients and of healthcare as a route into discussing research and finding out what was important to them. The issues raised by young people reflected the findings of research conducted over a decade ago [29] and more recent needs assessments [16].

Finally, although research priority setting has been used elsewhere, e.g. Joseph Lind Alliance approach, it remains unclear as to whether it was acted upon.

A strength of the study is the national youth panel being developed from this research [30] we are aiming to involve young people in research in a range of ways to ensure that a broad range of young people are involved in terms of age, gender, location, diagnosis, experience of illness and satisfaction with care. The first meeting of the group was held in October 2016 and an online involvement approach has been developed using social media.

Demonstrating cause and effect in terms of evaluating the impact that young people’s involvement has on future research strategies in this area is likely to be essential.

## Conclusions

Young people with rheumatic conditions have clear opinions on what should be researched in the rheumatology arena. Understanding the influences on these opinions will be key to both future involvement and research strategies. Future evaluation of the BANNAR national youth panel will aim to contribute further to this area of research.

## Abbreviations

BANNAR: Barbara ansell national network for adolescent rheumatology
BSPAR: British Society for Paediatric and Adolescent Rheumatology
CHAQ: Childhood health assessment questionnaire
GP: General practitioner (primary care doctor in the UK)
MAHSC: Manchester academic health sciences centre
NHS: National health service
PPI: Patient and public involvement
PSPs: Priority setting partnerships
YOURR: Young people’s opinions underpinning rheumatology research
